# E/e' Ratio as a Predictor of In-Hospital Complications and Clinical Outcomes of Acute Myocardial Infarction

**DOI:** 10.7759/cureus.66795

**Published:** 2024-08-13

**Authors:** Beka Mikeladze, Nino Zhvania, Giorgi Nikolaishvili

**Affiliations:** 1 School of Medicine, New Vision University, Tbilisi, GEO; 2 Pre-Clinical Department, Avicenna Batumi Medical University, Batumi, GEO; 3 Cardiology Department, Chapidze Emergency Cardiology Center, Tbilisi, GEO; 4 Clinical Department, Avicenna Batumi Medical University, Batumi, GEO; 5 Cardiology Department, High-Tech Hospital Medcenter, Batumi Referral Hospital, Batumi, GEO

**Keywords:** predictor, clinical outcomes, in-hospital complications, e/e' ratio, acute myocardial infarction

## Abstract

Introduction

Acute myocardial infarction (AMI) is the leading cause of death worldwide. E/e’ ratio can predict complications and mortality in the long term, but information on its effectiveness in in-hospital settings is limited. Identifying high-risk patients effectively is crucial for early management, which may improve overall clinical outcomes. This study evaluated the predictive value of the E/e' ratio of in-hospital complications and clinical outcomes of AMI.

Methods

Patients presenting with AMI to Batumi Referral Hospital, Georgia, between December 2023 and May 2024, were examined in this study. During the first 24 hours of hospitalization, all patients underwent two-dimensional echocardiograms. Early diastolic filling velocity (E) was measured by pulsed wave Doppler, and early mitral annulus velocity (e') was measured by tissue Doppler. Based on the ratio of the two measures, patients were divided into three groups: E/e'<8, E/e' 8-14, and E/e'>14.

Results

A total of 113 patients (92 males and 21 females) were included in the study. The average age of the patients was 62 years (SD = 11.71). Overall, 27 (23.8%) patients were diagnosed with ST-elevation myocardial infarction (STEMI) and 86 (76.2%) patients with non-ST-elevation myocardial infarction (NSTEMI). The E/e' ratio was normal (less than 8) in 39 (34.5%) patients (group A), increased to 8-14 in 46 (40.7%) (group B), and greater than 14 in 28 (24.8%) (group C). The percentage of deaths in group B was higher than that in group A (2.2% vs 0%), and in group C, it was significantly higher than that in group B (17.9% vs 2.2%) (p<0.05). The percentage of heart failure developed during hospitalization was higher in group B compared to group A (54.3% vs 2.6%), and it was significantly higher in group C compared to group B (67.9% vs 54.3%) (p<0.05). The percentage of arrhythmias developed during hospitalization was higher in group B compared to group A (13.0% VS 2.6%), and in group C, was significantly higher compared to group B (35.7% VS 13.0%) (p<0.05). The percentage of cardiogenic shock in group B was higher than that in group A (4.3% VS 0%), and in group C, it was significantly higher than group B (25.0% VS 4.3%) (p<0.05). No significant association was found between E/e' ratio and recurrent angina, nor was there a significant association between all-cause deterioration during hospitalization (p>0.05).

Conclusions

Both slightly and significantly elevated E/e' ratios are predictors of in-hospital complications and may be used to identify individuals at high risk of negative health outcomes.

## Introduction

The most severe manifestation of coronary artery disease (CAD) is acute myocardial infarction (AMI). Hospitalized patients diagnosed with AMI experience an in-hospital mortality rate of 7% and an overall five-year mortality rate of 19% [1]. Determining left ventricular diastolic dysfunction (LVDD) in patients with AMI is important for predicting mortality and development of heart failure (HF) [2-4]. Left ventricular filling pressure (LVFP) is a crucial indicator of diastolic dysfunction because it directly correlates with the ability of the heart to relax and fill with blood during diastole. [5] Until now, invasive cardiac catheterization, associated with high risks of complications, is considered the gold standard in assessing LVFP [6]. The most accurate noninvasive measurement of LVFP is the ratio of early diastolic mitral inflow velocity (E) to early diastolic mitral annular velocity (e') [7]. An E/e' ratio <8 is considered normal, while a ratio of >15 indicates an increase in LVFP [8]. Most studies on the impact of LVDD on AMI outcomes have focused on post-hospital complications and long-term outcomes, with limited information on the immediate in-hospital impact. This study investigated the correlation between the E/e' ratio and complications of AMI, duration of hospital stay, and clinical outcomes.

## Materials and methods

Study population

From December 2023 to May 2024, patients admitted to the Batumi Referral Hospital, who were diagnosed with AMI according to the European Society of Cardiology (ESC) criteria [9], were invited to participate in the study. Eligible participants were adults over the age of 18 with written consent to participate in the study. Patients were excluded if they had acute coronary syndrome that did not qualify as AMI, had a history of hypertrophic cardiomyopathy or severe left ventricular hypertrophy detected by transthoracic echocardiography, had severe mitral and/or aortic valve stenosis, had a history of confirmed and documented infiltrative cardiomyopathy, had acute or chronic infective endocarditis, had cardiac tamponade or constrictive pericarditis, or had any type of tachycardia that would interfere with echocardiographic assessment of diastolic function. Out of 134 patients, 113 met the criteria and agreed to participate in the study. The study received approval from the Ethical Committee of Batumi Referral Hospital (approval number: 2023-28-11-1; date: 28.11.2023) and adhered to the ethical standards and policies of the hospital.

Data collection

All patients underwent echocardiography after diagnosis of AMI. A selective coronary angiography was performed on the patient according to medical indication, immediately in the case of ST-elevation myocardial infarction (STEMI), and within 24 hours of hospitalization in the case of non-ST-elevation myocardial infarction (NSTEMI), thus confirming the etiology of the diagnosis, i.e., the presence of coronary atherosclerosis. During each patient's hospitalization, information was collected about age, sex, CAD risk factors, and concurrent diseases. Electrocardiogram and echocardiographic data were also collected and analyzed. In each case, at the end of hospitalization, information on in-hospital complications and outcomes was collected.

Echocardiographic evaluations

Two-dimensional echocardiographic evaluations were performed during the first 24 hours of hospitalization with an ultrasound system (Philips, Affiniti 70, serial number US716F0456) equipped with tissue Doppler imaging software and a variable frequency phase shifter from 2.5 to 5 MHz. Mitral flow patterns were assessed by pulsed wave Doppler in the apical four-chamber and two-chamber positions, and early (E) and late (A) diastolic filling velocities were measured. Tissue Doppler was also performed in the apical four-chamber position to measure early (e’) and late (a’) mitral annulus velocities. LVFP was calculated using the E/e' ratio. Patients were divided into three groups based on the E/e' ratio thresholds provided by the ESC [8]: group A (<8), group B (8-15), and group C (>15).

Complications and clinical outcome definition

According to the research protocol, complications of AMI included systemic (cardiogenic shock [CS], HF, embolic cerebrovascular event, and systemic and lower extremity embolism), mechanical (mitral valve and chordae rupture/tear, ventricular septal defect, ventricular free wall rupture, tamponade, aneurysm), ischemic (reinfarction, peri-infarct ischemia, infarct extension), arrhythmic (heart blocks, atrial and ventricular arrhythmias) and inflammatory (pericarditis, post-MI Dressler syndrome) events. As a complication, HF was defined as HF developed during hospitalization, which is classified as Killip class III.

Other complications included hospital-acquired infections and exacerbations of concurrent non-cardiac chronic conditions. Cardiac death was considered the clinical outcome.

Statistical analyses

The statistical data processing computer program SPSS Version 26 (IBM Corp., Armonk, NY) was used for the processing and analysis of the results. Based on the goals and objectives of the research, the data analysis was carried out using the following statistical methods: cross-tabulation analysis (Pearson's chi-squared test), residual statistics, and variance analysis was carried out to determine the differences between groups. Independent samples t-test and ANOVA were used to compare the variables among the groups. A p-value of <0.05 was considered statistically significant.

## Results

Clinical characteristics

The study included 113 patients (92 males and 21 females). The average age of the patients was 62 years (M = 62.06; SD = 11.71). Overall, 27 (23.8%) patients were diagnosed with STEMI and 86 (76.2%) patients with NSTEMI. Group A included 39 patients with normal LV filling pressure, group B included 46 patients with elevated values, and group C included 28 patients with significantly increased LVFP (Table 1).

**Table 1:** Baseline clinical characteristics CAD, coronary artery disease; HF, heart failure *Considered significant at a p-value of less than 0.05

Variable	Total (n=113)	Group A (n=39)	Group B (n=46)	Group C (n=28)	P-value
Male	92 (81.4%)	34 (87.1%)	37 (80.4%)	21 (75.0%)	0.439
Female	21 (18.6%)	5 (12.8%)	9 (19.5%)	7 (25.0%)	0.655
Age	62.0 ± 11.71	59.1±11.45	60.39±11.48	68.93±9.94	0.001*
Risk factors for CAD and concurrent diseases					
Dyslipidemia	30 (26.5%)	7 (17.9%)	16 (34.7%)	7 (25.0%)	0.211
Smoker	76 (67.3%)	28 (71.7%)	30 (65.2%)	18 (64.2%)	0.754
Alcohol consumption	61 (54.0%)	22 (56.4%)	23 (50.0%)	16 (57.1%)	0.779
Diabetes mellitus	33 (29.2%)	6 (15.3%)	18 (39.1%)	9 (32.1%)	0.052
Menopause	20 (95.2%)	5 (100.0%)	8 (88.8%)	7 (100.0%)	0.435
Hypertension	93 (82.3%)	29 (74.3%)	38 (82.6%)	26 (92.8%)	0.147
HF history	10 (8.8%)	0 (0%)	3 (6.5%)	7 (25.0%)	0.001*

Complications

During hospitalization, HF occurred in 45 (39.8%) of patients. Compared to patients in group A (n=1; 2.6%), group B had a significantly higher incidence of HF during hospitalization (n=25; 54.3%), and it was even more common in group C (n=19; 67.9 %), p<0.05. The rhythm abnormality was observed in 17 of the patients. Compared to patients in group A (n=1; 2.6%), significantly more patients in group B (n=6; 13%) and group C (n=10; 35.7%) experienced arrhythmia (p<0.05). In the case of CS, 8% of patients developed (n=9). In group A, CS was not observed at all. In group B, CS was detected in 4.3% of cases (n=2), and in group C more than that in group B, 25% (n=7) (p<0.05). No significant association was found between E/e' ratio and recurrent angina. Recurrent angina was found in 17.9% in group A, 28.3% in group B, and 25% in group C (p>0.05). However, taking into account that the normal E/e' ratio rate was spread only in group A, there is a considerable difference between normal (E/e'<8) and pathological (E/e'>8) ratio (A = 17.9% vs B and C = 53.3%) (Table 2). Mechanical, inflammatory, and ischemic complications were not detected in any patient. No cerebral or peripheral artery embolism was detected.

**Table 2:** In-hospital complications of AMI HF, heart failure; CS, cardiogenic shock; AMI, acute myocardial infarction *Considered significant at a p-value of less than 0.05

Variable	Total (n=113)	Group A (n=39)	Group B (n=46)	Group C (n=28)	P-value
HF	45 (39.8%)	1 (2.6%)	25 (54.3%)	19 (67.9%)	0.000*
Arrhythmia	17 (15.0%)	1 (2.6%)	6 (13.0%)	10 (35.7%)	0.001*
CS	9 (8.0%)	0 (0%)	2 (4.3%)	7 (25.0%)	0.000*
Recurrent angina	27 (23.9%)	7 (17.9%)	13 (28.3%)	7 (25.0%)	0.533

Outcomes

Six patients died during hospitalization. There were no cardiac deaths in group A, one (2.2%) death in group B, and five (17.9%) deaths in group C. Deterioration from any cause was observed in 28 patients: seven (17.9%) in group A, 13 (28.3%) in group B, and eight (28.6%) in group C. No relationship was found between E/e' and the worsening of any other condition (Table 3).

**Table 3:** AMI outcomes AMI, acute myocardial infarction *Considered significant at a p-value of less than 0.05

Variable	Total (n=113)	Group A (n=39)	Group B (n=46)	Group C (n=28)	P-value
Death	6 (5.3%)	0 (0%)	1 (2.2%)	5 (17.9%)	0.007*
Worsen any other condition	28 (24.8%)	7 (17.9%)	13 (28.3%)	8 (28.6%)	0.063

Duration of hospital stay

Statistically significant differences were not found between the groups in terms of the length of hospitalization. Although the average number of hospital days was highest in group C (M=4.89), it was lowest in group A (M=4.23) (Table 4).

**Table 4:** Duration of hospitalization stay after AMI AMI, acute myocardial infarction

Variable	Total (N=113), mean	Group A (n=39), mean	Group B (n=46), mean	Group C (n=28), mean	P-value
Day	4.47	4.23	4.41	4.89	0.652

## Discussion

In this study, we evaluated the predictive value of the E/e' ratio for in-hospital complications and clinical outcomes in patients with AMI. Our results demonstrated that both slightly and significantly elevated E/e' ratios are strong predictors of in-hospital complications, including HF, arrhythmias, and CS.

The findings indicate a clear association between higher E/e' ratios and adverse in-hospital outcomes. Patients with an E/e' ratio between 8 and 14 (group B) and those with an E/e' ratio greater than 14 (group C) had significantly higher incidences of HF, arrhythmias, and CS compared to those with a normal E/e' ratio (group A). Specifically, the mortality rate in group C was significantly higher than that in groups A and B, which underscoring the E/e' ratio’s potential as a valuable prognostic tool.

Our study extends the findings of prior research that have established the E/e' ratio as a reliable marker for diastolic dysfunction and a predictor of adverse outcomes in heart failure patients. Nagueh et al. [10] and Ommen et al. [11] highlighted the E/e' ratio's utility in predicting long-term outcomes. Our study, however, is among the first to emphasize its predictive value for in-hospital complications in AMI patients, addressing a critical gap in the literature.

Other studies, such as those by Moller et al. [12] and Hillis et al. [4], also support the use of echocardiographic parameters in predicting outcomes post-AMI. These studies predominantly focused on post-discharge prognosis, whereas our research underscores the immediate implications of elevated E/e' ratios during the hospitalization period, thereby providing actionable insights for acute care settings.

The significant association between elevated E/e' ratios and in-hospital complications suggests that early echocardiographic assessment can be instrumental in identifying high-risk AMI patients. This early identification allows for timely and aggressive intervention, potentially improving clinical outcomes. Incorporating the E/e' ratio into routine evaluation protocols for AMI patients could enhance risk stratification and guide therapeutic decisions.

For instance, early aggressive management strategies, as suggested by O'Gara et al. [13], could be tailored for patients with elevated E/e' ratios, potentially mitigating the risk of adverse outcomes. The integration of E/e' ratio measurements into standard clinical practice could facilitate more personalized treatment approaches, enhancing overall patient care.

Nallapati et al. [14] further corroborated these findings by demonstrating that elevated E/e' ratios are associated with poor functional outcomes and increased mortality in AMI patients. Their study also emphasized the utility of the E/e' ratio as a non-invasive measure to guide clinical decision-making in the acute setting. Similarly, Santos [15] found that higher E/e' ratios were predictive of adverse cardiovascular events and were effective in stratifying risk among AMI patients, supporting the integration of E/e' ratio assessment into routine clinical practice.

There are several limitations to our study. Firstly, the sample size was relatively small and drawn from a single center, which may limit the generalizability of our findings. Secondly, the study’s observational nature does not establish causality. Future studies with larger, multicenter cohorts and randomized designs are needed to validate our results and establish causative relationships.

Additionally, our study did not account for all potential confounding factors, such as the presence of other comorbidities and variations in treatment protocols. Addressing these factors in future research could provide a more nuanced understanding of the E/e' ratio’s predictive value.

Further research should explore the utility of the E/e' ratio in different populations and settings and post-discharge scenarios. Additionally, investigating the combination of the E/e' ratio with other biomarkers could provide a more comprehensive risk assessment tool. Studies focusing on interventions targeted at patients with elevated E/e' ratios could also offer insights into improving management strategies for this high-risk group.

Moreover, longitudinal studies examining the long-term outcomes of patients stratified by E/e' ratio at admission could elucidate the extended prognostic value of this parameter. Such research could potentially lead to the development of more robust, evidence-based guidelines for managing AMI patients with varying E/e' ratios.

## Conclusions

Our study demonstrates that the E/e' ratio is a valuable predictor of in-hospital complications in AMI patients. HF, arrhythmias, and CS were strongly associated with elevated E/e' ratios. These results show the importance of early echocardiographic assessment in identifying high-risk patients, thus improving clinical outcomes. This proactive approach may reduce mortality and morbidity.

According to our findings, the E/e' ratio can be an essential tool in the early intervention of AMI. Future studies should validate these findings in larger, diverse populations and explore the combination of the E/e' ratio with other biomarkers for comprehensive risk assessment. This will enhance our ability to manage LVDD in AMI patients and improve patient care.
